# Rapid Spread and Genetic Characterisation of a Recently Emerged Recombinant Lumpy Skin Disease Virus in Thailand

**DOI:** 10.3390/vetsci9100542

**Published:** 2022-09-30

**Authors:** Nutthakarn Suwankitwat, Tapanut Songkasupa, Prakit Boonpornprasert, Phurida Sripipattanakul, Sirin Theerawatanasirikul, Taweewat Deemagarn, Minta Suwannaboon, Orapun Arjkumpa, Noppawan Buamithup, Akkarapol Hongsawat, Sirima Jindajang, Nawakarn Nipaeng, Dilok Aunpomma, Lamul Molee, Kanokwan Puangjinda, Walaiporn Lohlamoh, Bandit Nuansrichay, Rawint Narawongsanont, Pipat Arunvipas, Porntippa Lekcharoensuk

**Affiliations:** 1 Department of Microbiology and Immunology, Faculty of Veterinary Medicine, Kasetsart University, Bangkok 10900, Thailand; 2 Department of Livestock Development, Bangkok 10400, Thailand; 3 Animal Health Section, The 4th Regional Livestock Office, Department of Livestock Development, Khon Kaen 40260, Thailand; 4 Bureau of Disease Control and Veterinary Services, Department of Livestock Development, Bangkok 10400, Thailand; 5 Veterinary Research and Development Center (Lower Northeastern Region), Department of Livestock Development, Surin 32000, Thailand; 6 Department of Large Animal and Wildlife Clinical Sciences, Faculty of Veterinary Medicine, Kamphaeng Saen Campus, Kasetsart University, Nakhon Pathom 73140, Thailand

**Keywords:** lumpy skin disease virus, outbreaks, diagnoses and examinations, genetic characterisation, the Kingdom of Thailand

## Abstract

**Simple Summary:**

Lumpy skin disease (LSD) is an economically important disease of cattle caused by LSD virus (LSDV), a member of poxviruses. It had never been found in Thailand before March 2021, but has since spread broadly to various provinces. Regional veterinarians have collected samples from the LSD cattle and submitted them for diagnosis as a part of disease surveillance during the outbreaks. Our study aimed to monitor the distribution of the outbreaks by recording the LSD cases based on clinical signs and laboratory tests up to June 2022, and characterise the causative agent virologically and genetically. Outbreak maps were created to illustrate the rapid temporal distribution of the LSD index cases in each province of Thailand. We detected two distant origins of the outbreaks. LSDV DNA was confirmed in blood, milk, and skin samples collected from sick animals by real-time PCR. LSDV was proven to be the causative virus based on serological, virological, and pathological diagnoses. By genetic analysis, the Thai LSDV is a recombinant virus derived from a vaccine strain previously appearing in China and Vietnam. Its genetic material is a mosaic hybrid genome containing the vaccine virus DNA as the backbone interspersed with DNA fragments of a field strain.

**Abstract:**

The emergence of the lumpy skin disease virus (LSDV) was first detected in north-eastern Thailand in March 2021. Since then, the abrupt increase of LSD cases was observed throughout the country as outbreaks have spread rapidly to 64 out of a total of 77 provinces within four months. Blood, milk, and nodular skin samples collected from affected animals have been diagnosed by real-time PCR targeting the *p32* gene. LSDV was isolated by primary lamb testis (PLT) cells, followed by Madin-Darby bovine kidney (MDBK) cells, and confirmed by immunoperoxidase monolayer assay (IPMA). Histopathology and immunohistochemistry (IHC) of a skin lesion showed inclusion bodies in keratinocytes and skin epithelial cells. Phylogenetic analyses of *RPO30* and *GPCR* genes, and the whole genome revealed that Thai viruses were closely related to the vaccine-derived recombinant LSDV strains found previously in China and Vietnam. Recombination analysis confirmed that the Thai LSDV possesses a mosaic hybrid genome containing the vaccine virus DNA as the backbone and a field strain DNA as the minor donor. This is an inclusive report on the disease distributions, complete diagnoses, and genetic characterisation of LSDV during the first wave of LSD outbreaks in Thailand.

## 1. Introduction

Lumpy skin disease (LSD) of cattle and water buffalo is characterised by the presence of fever, flat disc-like skin nodules on the body, and lymph node enlargement [1,2]. It is caused by the lumpy skin disease virus (LSDV), which belongs to the genus *Capripoxvirus* (CaPV), subfamily *Chordopoxvirinae,* family *Poxviridae*. LSDV is genetically related to the sheeppox virus (SPPV) and goatpox virus (GTPV) [3]. The virion is enveloped and contains a covalently cross-linked, double-stranded DNA genome of approximately 151 kb. The genome comprises identical 2.4 kb-inverted terminal repeats at both ends and a central sequence of 156 open reading frames (ORFs) coding for polypeptides of 53 to 2025 amino acids [4]. The virus particle possesses at least 30 structural proteins associated with virion morphogenesis and assembly [4].

LSD spreads rapidly and can cause significant economic losses, which must be reported to the World Organisation for Animal Health (WOAH) [5]. It was first reported in Zambia in 1929 [6] and expanded to several regions in Africa [7,8]. LSD has been found in the Middle East since 2012 [9] and in Southeast Europe and North Caucasus since 2015 [10,11]. In 2019, the disease was identified in Bangladesh, India, and China before spreading to other regions of Asia including Vietnam and Myanmar in 2020 [12,13,14,15,16]. The disease is transmitted predominantly by bloodsucking insects such as stable flies, ticks, and mosquitoes [17,18,19]. However, indirect contact transmission was recently demonstrated for a virulent, vaccine-derived recombinant LSDV strain [20]. Moreover, uncontrolled LSD-infected cattle through the legal and illegal movement of animals also play a role in disease transmission. In Thailand, the first LSD case appeared in Roi Et, the north-eastern province on 29 March 2021 [21]. The disease was detected by clinical signs and confirmed by real-time PCR targeting the *p32* gene as well as DNA sequencing of the fusion, *RPO30*, *GPCR*, and *ANK* gene regions [21,22,23]. From June 2021 onward, live attenuated vaccines including Lumpyvax™ (MSD, Pretoria, South Africa) and MEVAC™ (Kemin, Cairo, Egypt) were applied to control disease outbreaks in Thailand. There were 5,360,000 doses of imported vaccines, including 360,000 doses of Lumpyvax^TM^ and 5,000,000 doses of Mevac^TM^. However, there were about 9.9 million cattle in the country. Thus, the initial 360,000 ring vaccine doses were administered to disease-free areas and at-risk animals within a 5–50-km radius of epidemic areas in the north and northeast regions, respectively. Five million doses were used for widespread immunisation in all regions, with disease-free areas, livestock within a 5-km radius of national parks, and healthy animals on outbreak farms receiving priority.

Although extensive vaccination campaigns implemented on the total cattle population of a country is a keystone of disease eradication, successful LSD control and prevention also rely on early outbreak detection and rapid diagnosis [24]. Thus, this study aims to describe the diagnosis and distribution of LSD in Thailand and genetically characterise the circulating LSDV during the initial outbreak.

## 2. Materials and Methods

### 2.1. Sample Collection

Cattle and buffaloes exhibiting clinical signs such as skin nodules, enlarged lymph nodes, and fever were defined as suspected LSD cases, which were recorded on the sample submission form. From March to July 2021, samples from 859 suspected animals at 530 farms were submitted to the National Institute of Animal Health (NIAH), Department of Livestock Development (DLD), Thailand, for diagnosis. Concurrently, a total of 841 blood samples, 324 sera, 210 skin nodules, 18 organs, 8 milk samples, and 5 nasal swabs were collected from animals suspected of LSD. One LSDV-infected skin nodule in good condition with complete animal history was selected for histopathological [25] and IHC studies [26,27]. The clarified fluids from nodular skin tissue homogenates, blood, and milk were frozen at −80 °C for real-time PCR and/or virus isolation.

### 2.2. Geographical Mapping of the LSD Outbreaks

Index cases for each province were retrieved from the DLD E-smart surveillance system as secondary data. The cases were ordered by the date of reported clinical signs from March to June 2022. The data included all diseased animals matching the LSD case definition, positive by real-time PCR, or seropositive by ID Screen^®^ Capripox Double Antigen Multi-species ELISA (IDVET, Grabels, France) before vaccination. Outbreak maps were produced using QGIS software version 3.4.9 (QGIS.org, 2019) to illustrate the temporal distribution of the LSD index cases in each province per month. An animation of the outbreak distribution was performed by a 3D map in Excel.

### 2.3. Real-Time PCR

DNA was extracted from tissue homogenates, whole blood, and milk using a High Pure PCR Template Preparation Kit (Roche Diagnostics GmbH, Mannheim, Germany) according to the manufacturer’s instructions. The samples were initially screened by *p32* real-time PCR in a 20 µL reaction containing 4 nM of each *p32* specific primer [28] (Table S1), 1.2 nM of TaqMan probe, 5 µL of DNA template, 3.96 µL H_2_O and 10 µL of FastStart Essential DNA Probes Master (Roche Diagnostics GmbH, Mannheim, Germany). Thermal cycler conditions included initial denaturation at 95 °C for 10 min followed by 40 amplification cycles (95 °C for 15 s and at 60 °C for 45 s). The LSDV DNA levels were reported as threshold cycle (*Ct*) values. 

### 2.4. Virus Isolation and Detection

In this case, 15 skin samples and one milk sample positive by real-time PCR with the *Ct* less than 25 were selected for virus isolation. LSDV isolation was performed according to the OIE manual with some modifications [24,29,30]. Briefly, 10% skin tissue suspension and a 10-fold diluted milk sample were filtered through 0.45 µm. The processed samples were inoculated onto primary lamb testis (PLT) cells, which were incubated at 37 °C with 5% CO_2_ for four to nine days. Next, the virus isolates were harvested by three freeze-thaw cycles, clarified by centrifugation, and subsequently inoculated onto the PLT cells again. The LSDV isolates were further inoculated onto the Madin-Darby bovine kidney (MDBK) cell line (CCL-22™ Lot 63990115 ATCC^®^, Manassas, VA, USA). Once the CPE was observed, the virus was collected, aliquoted, and kept at −80 °C for real-time PCR. In parallel, the infected MDBK cells were fixed with 4% formalin buffer and stained with 0.15% crystal violet (Sigma-Aldrich, Hamburg, Germany). To detect viral proteins, LSDV was inoculated onto MDBK cells in a 96-well plate for immunoperoxidase monolayer assay (IPMA), as described elsewhere [31,32]. 

### 2.5. DNA Sequencing and Phylogenetic Analysis

Conventional PCR and DNA sequencing of the *RPO30* and *GPCR* genes were performed with specific primers (Table S1) on five selected qPCR skin positive samples including the first index case and representatives from each geographical region. The genes were amplified as described elsewhere [33] using GoTag^®^ Green Master Mix (Promega, USA) according to the manufacturer’s instructions. The PCR products were submitted for SANGER sequencing (Macrogen, Korea).

For the whole-genome sequencing (WGS), LSDV genomic DNA was extracted from a skin homogenate using the DNeasy blood and tissue kit (Qiagen, Hilden, Germany), according to the manufacturer’s instructions. The DNA library was constructed using the Nextera XT DNA library preparation kit. Sequencing was performed using a MiSeq reagent kit version 3 with 2 × 300-bp paired-end sequencing on a MiSeq benchtop sequencer (Illumina, San Diego, CA, USA). The quality of raw data was assessed using FastQC software version 0.11.9 (https://www.bioinformatics.babraham.ac.uk/projects/fastqc/ (accessed on 18 July 2021)). Genome assembly and annotation were performed using Geneious Prime software version 2021.2.2 (Biomatters Ltd., Auckland, New Zealand). The reads were trimmed using a BBDuk Trimmer based on quality (Q score > 30) and length (>20 bp). The trimmed reads were *de novo* assembled into contigs using SPAdes assembler version 3.15.2 and mapped to the reference genome (MZ577076). The full genome sequence of LSDV/Thailand/Yasothon/2021 was deposited in the GenBank database (OM033705).

The processed study sequences were compared and aligned with those of 36 CaPV strains (Table S2) using the Geneious Prime. The multiple sequence alignment and analyses of sequence identities and divergences were performed by MUSCLE 3.8.425, which is embedded in the Geneious Prime^®^ 2021.2.2. Phylogenetic trees of *RPO30*, *GPCR*, and WGS were constructed using the UPGMA method [34] exploiting the Tamura 3-parameter model [35] in MEGA 7 software version 7.0.26 [36] with 1000 bootstrap replicates [37] and default settings.

### 2.6. Recombination Analysis

Three LSDV genomic sequences including Yasothon/2021 (OM033705), SIS-Lumpyvax vaccine (KX764643), and Neethling 2490 field strains (AF325528) were aligned and analysed using the Recombination Detection Program (RDP) [38], Bootscan [39], MaxChi [40], GENECONV [41], Chimaera [42], and SiScan [43] available in the RDP software package version 4.101. Phylogenetic trees of recombination donor and acceptor (backbone) sequences and the corresponding regions of 16 LSDV reference strains (Table S2) were generated in RDP4 software using the Neighbour-Joining method with 100 bootstraps. Next, the potential recombination events were analysed by SimPlot software version 3.5.1 [44].

### 2.7. Statistical Analysis

Data were managed and analysed using Excel and GraphPad Prism software version 9.3.1. Briefly, the data were sorted by month, province, region, number of cattle, and qPCR results, respectively. All LSD-affected provinces in each region were summarized by month. Per cent of LSDV positive provinces in each region and month were analysed by Fisher’s exact test. Real-time PCR results from 111 animals’ skin and blood samples were statistically analysed using Chi-square and odds ratio to determine the likelihood of viral genome detection.

## 3. Results

### 3.1. Clinical Cases and Histopathological Findings

**Figure 1:**
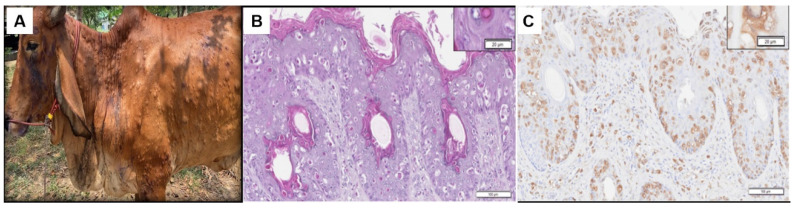
Clinical signs and histopathological examination of LSD-infected animals. (**A**) Skin nodules all over the body surface of a beef cattle. The nodules are 1–3 cm in diameter, circumscribed, round, and slightly raised. (**B**) Haematoxylin and Eosin (H&E) staining histopathological lesions demonstrating epidermal hyperplasia with hyper-eosinophilic cytoplasm and large numbers of spongiotic keratinocytes containing large, round eosinophilic intracytoplasmic inclusion bodies were found dispersed throughout the affected skin. The dermis and hypodermis were infiltrated by inflammatory cells. Moderate to severe dermal vasculitis and perivascular accumulation of inflammatory cells and fibrins were observed. The high magnification views (dashed boxes) depict the epidermis. (**C**) IHC of the corresponding areas in the consecutive tissue section is shown in B. The brown staining of LSDV antigens accumulated in the cytoplasm of the infected cells is shown in the inset.

All LSD suspected cases showed clinical signs including skin nodules, lymphadenopathy, and fever. The lumps appeared on the skin of the head, neck, body, and perineum (Figure 1A and Figure S1). Histopathological skin lesions were observed from the epidermis to deep dermis layers (Figure 1B and Figure S2), as previously reported [45]. The epidermis contained hydropic degeneration and oedema with basophilic nuclei and necrotic keratinocytes. Large numbers of spongiotic keratinocytes containing large, round eosinophilic intracytoplasmic inclusion bodies were found dispersed throughout the epidermis and dermis. The IHC of the consecutive slide revealed the brown cytoplasmic staining of LSDV antigens in the infected cells (Figure 1C and Figure S3).

### 3.2. Distribution of LSD

**Figure 2:**
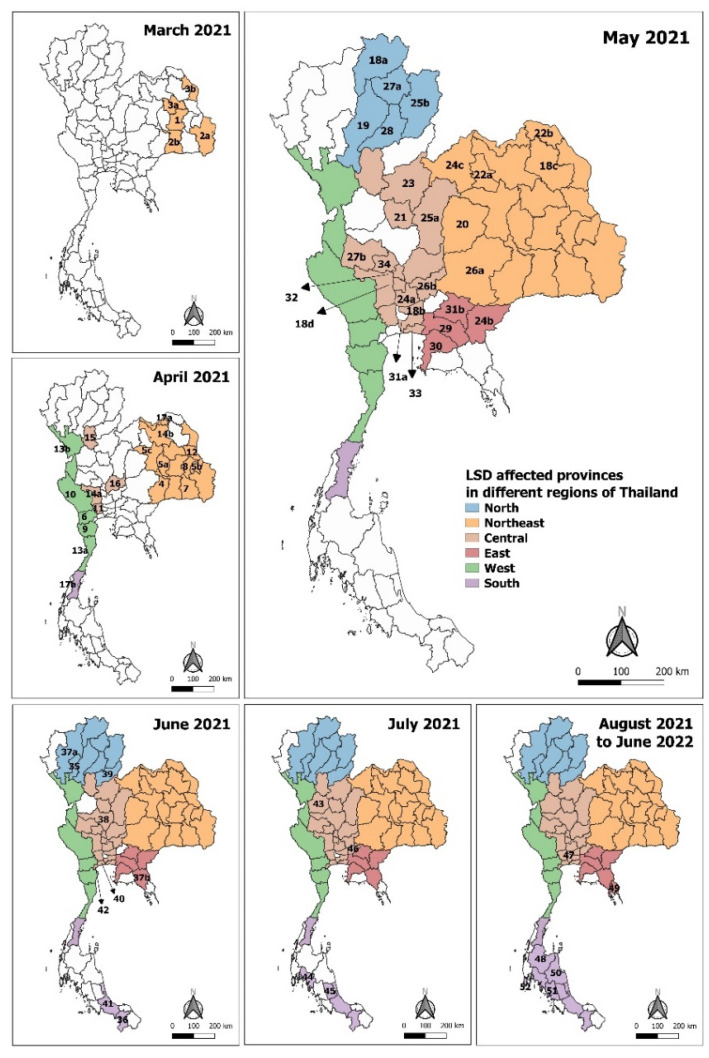
Distribution of LSD outbreaks in Thailand from March 2021 to June 2022. The affected provinces were mapped, and assorted colours were assigned for each geographical region of Thailand: The Northeast (orange), Central (brown), West (green), East (red), North (blue), and South (purple) using QGIS software (QGIS.org, 2019). The numbers represent provinces with LSD cases ordered by outbreak timelines. The names of provinces are displayed in Table S3.

**Figure 3:**
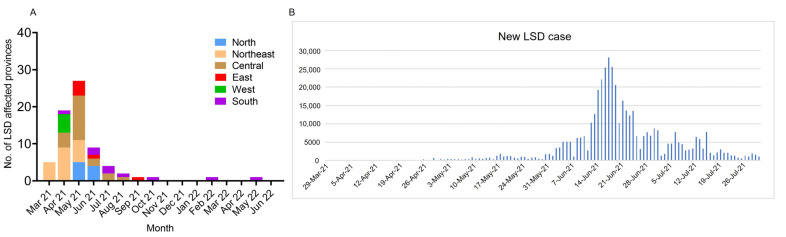
Number of reported LSD-affected provinces and cases during the first epidemics in Thailand. (**A**) The bar chart shows the numbers of LSD-affected provinces from March 2021 to June 2022 in each geographical region of Thailand. (**B**) The epidemic curve of new LSD cases from March to July 2021. Data were retrieved from E-smart surveillance (DLD, 30 June 2022).

Since the confirmed case in north-eastern Thailand in March 2021 [21], we investigated and collected data on LSD cases based on clinical signs, real-time PCR, and serological tests up until June 2022 via the E-smart Surveillance system. There were 628,089 affected animals and 283,212 affected farmers (data retrieved from DLD on 30 June 2022). During the first five months of the outbreak from March to July 2021, the percentage of LSD-positive provinces varied dramatically by month (*p* < 0.0001). The disease spread from the first outbreak in the northeast to four nearby provinces in three days. In April, the number of LSD-affected provinces increased considerably (*p* < 0.01), as LSD expanded to nine adjacent provinces. Concurrently, new outbreaks distant from the events in the northeast occurred in 10 provinces in central, western and southern Thailand (Figure 2, Tables S3 and S4, and Video S1). In May, the outbreaks expanded to 27 other provinces in four regions (Figure 3A, Table S4). The number of newly-affected provinces decreased remarkably in June (*p* < 0.01) and July (*p* < 0.00001) to nine and four provinces, respectively. However, the number of new LSD cases reached a peak in June with almost 30,000 reported cases per day, and then reduced significantly in July (Figure 3B), corresponding to the vaccination campaign established in June 2021. From August 2021 to June 2022, six provinces were affected by the disease, four of which were in the southern region (Figure 3A, Table S4). There were a few new cases during these 11 months (Table S3). In this wave, LSD spread to a total of 70 provinces in six regions of Thailand.

### 3.3. LSDV Diagnosis

From March to July 2021, 426 out of 859 LSD suspected animals were confirmed positive by *p32* real-time PCR (data not shown). To determine the likelihood of viral genome detection, skin and blood samples collected at the same time from every 111 animals were tested by real-time PCR, as shown in Table 1. The quantity of LSDV-positive skin was significantly higher than blood samples (*p* < 0.0001) with an odds ratio of 10.18.

**Figure 4:**
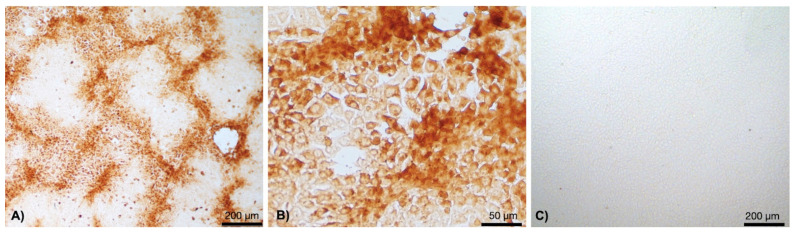
IPMA of LSDV infected MDBK cells. The cells were incubated with LSDV-specific antibodies in the convalescent bovine serum, followed by HRP conjugated bovine IgG, and stained with DAB substrate. LSDV infected cells contained brown staining LSDV antigens predominantly in the cytoplasm. Cytopathic effects (CPE) appeared as groups of aggregated LSDV infected cells (**A**,**B**). Non-infected cells remained unstained and served as a negative control (**C**).

From the 15 selected positive samples, LSDV could be isolated from four skin samples and one milk sample, collected seven days after the presence of skin nodules. After three passages in the cell culture, the aggregated cell foci with IPMA-positive viral antigens in the cytoplasm were observed at three days post-infection (dpi) (Figure 4A,B and Figure S4). Non-infected cell control remained CPE and IPMA negative (Figure 4C).

### 3.4. DNA Sequencing and Phylogenetic Analysis

The sequences for *RPO30* and *GPCR*, as well as the whole genome of Thai LSDV, were submitted to the GenBank database with the accession numbers shown in Table S5. Both the *RPO30* and *GPCR* genes of five Thai LSDVs were 100% identical. Phylogenetic analysis of the *GPCR* gene revealed two main subgroups including virulent field viruses of subgroup I and attenuated vaccine strains of subgroup II (Figure S5A), and the Thai sequences were clustered with the field strains. Additionally, the *RPO30* genes were phylogenetically divided into three subgroups (I-III), and the Thai sequences were grouped with the vaccine strains (Figure S5B). 

**Figure 5:**
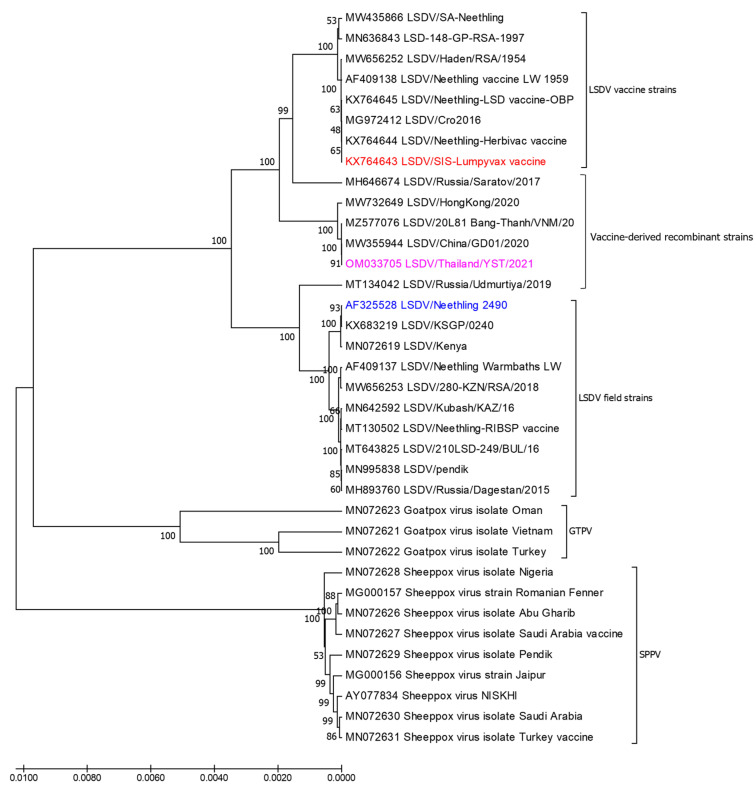
Phylogenetic tree based on the whole genome sequences of Thai LSDV (pink) and 35 reference strains, including CaPV. SIS-Lumpyvax vaccine (red) and Neethling 2490 field virus (blue) were used in recombinant analysis. The trees were constructed by the UPGMA method with 1000 bootstraps. The evolutionary distances were computed using the Tamura 3-parameter model. Evolutionary analyses were conducted in MEGA 7.

The whole genome of LSDV/Thailand/Yasothon/2021 consisted of 150,689 nucleotides, with 156 predicted protein-coding genes. The total number of reads was 3,752,850 and the depth of coverage was 31.1×. Phylogenetic analysis among 35 LSDV reference strains showed that Yasothon/2021 clustered between the vaccine and field groups, and closely related to the recombinant viruses (Figure 5). Nucleotide identities between field LSDVs and the Thai isolate, vaccine strain or wildtype virus are presented in Table S2. Thai LSDV is nearly identical to the Vietnamese LSDV with two SNPs and one InDel within three coding regions of genes, including the ORF071 C62,252T, ORF103 C98,430T, and ORF144 T136,995del in the genome. This phenomenon caused amino acid changes in the ORF103 E/K encoding virion core protein as well as amino acid changes with three insertions in the ORF144 VFFVKT/VFL encoding Kelch-like protein. Compared to the SIS-Lumpyvax vaccine, Thai LSDV had five frameshifts in the ORF086, ORF087, ORF131, ORF134, and ORF144.

### 3.5. Recombination Analysis

**Figure 6:**
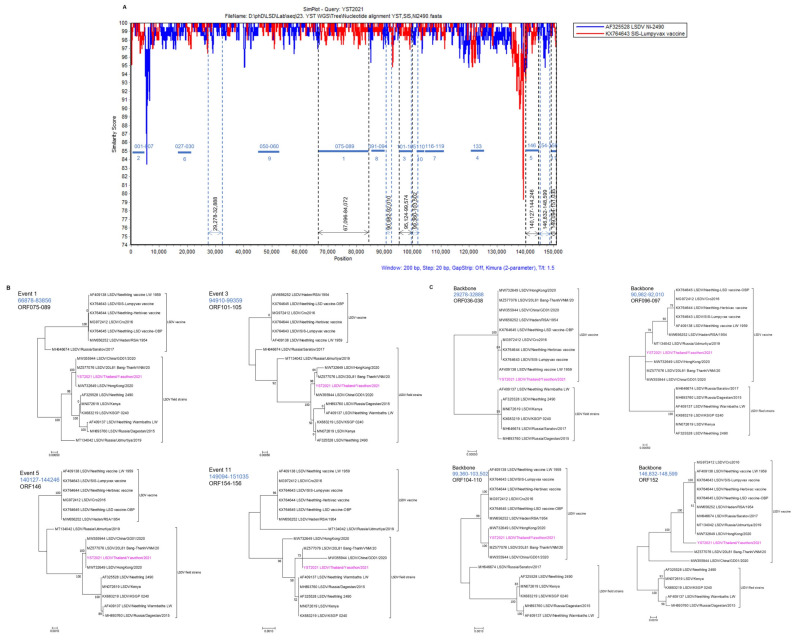
Recombination analysis of the LSDV genome using SimPlot. (**A**) Sequence similarity of the LSDV/Yasothon/2021 was compared to those of the SIS-Lumpyvax vaccine strain (in red) and the field strain NI-2490 (in bright blue). The thick dark-blue horizontal lines represent the positions of the predicted recombination events. The numbers on the horizontal lines are the ORF numbers. The numbers under the horizontal lines are the order of the recombination events. Eight regions selected for the phylogenetic analyses were marked by arrows with the nucleotide sequence positions. (**B**,**C**) Phylogenetic trees for the donor regions of 4 recombination events and 4 selected backbone sequences, respectively. Blue numbers indicate the sequence positions used for the phylogenetic analyses, and the LSDV/Yasothon/2021 genome is pink. The phylogenetic tree construction and evolutionary analyses were performed as described in the method. Recombination identification and analysis were conducted in RDP software package version 4.101.

**Figure 7:**
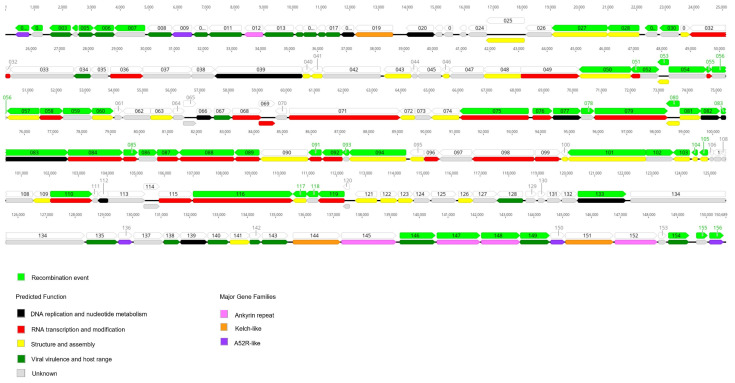
Gene mapping of LSDV/Thailand/Yasothon/2021. The arrows indicate transcriptional directions. The numbers represent the nucleotide positions from 1 to 150,689 bp. Each ORF is colored based on its recombination event (upper), predicted protein function, and major gene families (lower) [3]. The graphic was created using Geneious Prime^®^ version 2021.2.2. Light green = recombination events; Black = DNA replication and nucleotide metabolism; Red = RNA transcription and modification; Yellow = structure and assembly; Dark green = viral virulence and host range; Grey = unknown; Pink = Ankyrin repeat; Orange = Kelch-like; Purple = A52R-like.

Recombination analysis of Yasothon/2021 with the parental strains, SIS-Lumpyvax vaccine and Neethling 2490 field virus revealed 11 recombination events in the 59 ORFs within the Thai LSDV genome (Table 2). All potential recombinations were confirmed by a similarity plot (Figure 6A). Phylogenetic analysis of the Thai LSDV and 16 reference strains suggested that Yasothon/2021 is a vaccine-derived, recombinant virus. Thai LSDV was clustered with the field viruses in the trees constructed from four selected recombination events (1, 3, 5, and 11), while it was in the vaccine group in the backbone sequence trees (Figure 6B,C). The 11 recombination events hit 42 proteins in which 11 proteins were involved with RNA transcription and modification, nine proteins associated with viral structure and assembly, six proteins for viral virulence and host range, three proteins for DNA replication and nucleotide metabolism, two proteins were Ankyrin repeat, two proteins were A52R-like protein, and nine proteins were unclear function (Figure 7, Table 3). Interestingly, the changes of 13 proteins in the Thai LSDV genome have never been reported in the China/GD01/2020 and Russian/2017 strains, such as IL-10 (ORF005), EEV (ORF027), RNA polymerase subunit (ORF116 and ORF199), DNA helicase (ORF110), and apoptosis regulator (ORF154).

## 4. Discussion

This study found severe clinical signs of LSD in naive young and old animals, with lesions of the eyes, legs, and internal organs, possibly due to being immunocompromised. The outbreaks dissimilated rapidly throughout the country within three months. The first case occurred in the northeast [21], while the second appeared distantly in the west. Subsequently, the outbreaks were distributed from both epidemic centres to the central, southern, northern, and eastern regions. As the distance between north-eastern and western provinces is more than 500 km, the spread might have been facilitated by the movement of infected animals [46,47]. The number of affected provinces increased in April, Thailand’s summer season, possibly due to an increase in insect populations. The number of LSD-affected provinces decreased significantly from June to July 2021, possibly due to the vaccination campaign. This result was consistent with previous reports that vaccination is key to controlling the disease [48,49]. Since the imported vaccine quantities were insufficient to cover 80 per cent of the cattle population, however, there were occasional outbreaks in the southern region during February and May 2022, when the animals were new-born calves that had not been immunised.

For real-time PCR diagnosis, the skin nodules may be the most suitable sample as they harboured live viruses for up to 35 days and viral nucleic acids for up to 3 months [50], while LSDV could be detected intermittently in blood during 7–21 dpi [51]. Thus, negative blood samples did not infer an infection-free status. We suggested collecting skin lesions instead of blood during the intense outbreaks for the reasons stated above, as well as the advantages of a non-invasive approach and minimal equipment required. However, we were unable to control the sample collection process in order to acquire day-after symptoms that should be examined further.

Previously, LSDV and its genetic diversity were determined based on the fusion gene sequence [21,52]. Furthermore, vaccine and field strains were differentiated by *RPO30* and *GPCR* real-time PCRs (DIVA). In our phylogenetic trees, *RPO30* and *GPCR* of Thai LSDV were clustered in separated groups, similar to a previous study in Bangladesh [15]. We have shown that the Thai viruses were close to the vaccine strain based on the *RPO30* genes while they were clustered with the field strains in the *GPCR* tree. Thus, it is necessary to use more than one locus for investigating the potential recombination [53]. Our whole genome analysis suggests ORF006, ORF133, ORF146, and ORF148 as the alternative DIVA target genes. 

Whole genome sequencing revealed that ORF027 encoding EEV glycoprotein of Thai LSDV is similar to those of Vietnamese, but differs slightly from the Chinese and Russian strains [53,54]. Furthermore, the Thai LSDV contains the Kelch-like protein, which is a virulence determinant [55]. Lambs inoculated with a mutant SPPV including a deletion in the Kelch-like coding gene exhibited a marked reduction or delay in pyrexia, gross lesions, viremia, and virus shedding compared to the parental and revertant viruses [55]. In comparison to the Vietnamese strain, Thai LSDV has three extra amino acids in the protein, which might be associated with the virulent phenotype. Moreover, the five frameshifts found in Yasothon/2021 were consistent with LSDV/China/GD01/2020 [56]. All of these changes in the structural and non-structural proteins might contribute to the rapid spread of the virus in terms of infectivity and virulence. For example, the recombinant strain can spread by indirect contact such as by sharing food and water troughs, as well as physical touch with nearby animals [20]. The infected animals may exhibit viremia and shed the virus through nasal and oral discharge for up to 38 days after infection, which is much longer than previously reported by Babiuk et al. [57]. 

Up to now, the recombinant vaccine strains have been reported in Russia [53,54], China [56,58,59], and Vietnam [14,16]. According to the timeline of the LSD outbreak in Asian countries described previously [16,60], our findings indicated that viral genetics mutated slightly as the virus spread to Thailand. Only 13 out of 156 proteins of LSDV/Thai/2021 were changed from Chinese/2020 strains and two proteins were changed from the Vietnamese/2020 strain. A recent study divided recombinant strains into four groups (R1-R4), with all Asian strains falling within R4 [61]. These novel strains were most likely the result of a spillover from animals vaccinated with the Lumpivax vaccine (Kevevapi, Kenya), which was widely used in Kazakhstan shortly before the emergence of the vaccine-like strains in Russia [61]. Lumpivax vaccine is composed of at least three CaPVs, namely the Neethling-like LSDV vaccine strain or Herbivac, the KSGP-like LSDV vaccine strain, and the Sudan-like GTPV strain [61]. This outcome was similar to a prior study by Haegeman et al. [62] and stressed the need for vaccine quality control. The Thai LSDV acquired a major backbone from the SIS-Lumpyvax, which was genetically identical to Herbivac. The minor fragment derived from the NI-2490 strain was closely related to the KSGP strain. As a result, Thai LSDV might be in the R4 group with other Asian strains. 

Thai isolates were nearly identical to the Vietnamese and Chinese strains, suggesting the same origin. The common route of cattle movement in the Greater Mekong subregion could be the potential pathway for LSD transmission between southern China and Southeast Asia [63,64]. Another migration track could be from Myanmar to the western provinces of Thailand before heading north to Chiang Rai and south to Malaysia [65]. Although cattle movement and illegal trade are considered the main source of virus transmission between regions and countries, insect vectors may have been responsible for the short-distance spread (usually < 50 km) [5,60,66]. As a result, cattle movement and the strengthening of insect control programmes must be strictly regulated in order to minimise the spread of the epidemic in the country as well as neighbouring countries.

## 5. Conclusions

This work reported on rapid distributions and comprehensive LSD diagnoses in Thailand. This study exhibited extensive detection methods for Thai LSDVs as well as genetic characterisation. It also revealed the close relationship between Chinese, Vietnamese, and Thai LSDVs based on the *RPO30* and *GPCR* genes, and the whole genome. Further studies on epidemiology may elucidate the source of the outbreak and the spread pattern of LSDV in Thailand.

## Figures and Tables

**Table 1:** Results of *p32* real-time PCR for skin and blood samples collected from 111 animals.

	Skin	Blood	Total
**Positive**	103	62	165
**Negative**	8	49	57
**Total**	111	111	222

**Table 2:** Predicted recombination events determined by different detection methods available in the RDP4 program.

Recombination Event Number	Breakpoint Positions in Alignment		Size (bp)	ORF	Detection Methods						
	Begin	End			RDP	GENECONV	Bootscan	Maxchi	Chimaera	SiScan	3Seq
1	67,096	84,072	16,977	075–089	NS	4.95 × 10^−35^	4.27 × 10^−20^	2.30 × 10^−16^	4.98 × 10^−17^	NS	2.22 × 10^−16^
2	22 *	4792	4771	001–007	NS	2.01 × 10^−18^	1.31 × 10^−16^	2.80 × 10^−15^	2.80 × 10^−15^	NS	NS
3	95,124 *	99,574	4451	101–105	NS	1.19 × 10^−16^	1.13 × 10^−16^	4.94 × 10^−10^	4.94 × 10^−10^	NS	5.24 × 10^−14^
4	120,668 *	122,753	2086	133	NS	1.95 × 10^−17^	4.66 × 10^−20^	8.64 × 10^−13^	8.64 × 10^−13^	NS	1.67 × 10^−15^
5	140,127	144,246	4120	146–149	3.41 × 10^−7^	7.09 × 10^−13^	1.97 × 10^−9^	1.24 × 10^−14^	1.24 × 10^−14^	NS	5.55 × 10^−15^
6	19,579	23,622	4044	027–030	NS	2.54 × 10^−14^	1.34 × 10^−15^	1.29 × 10^−8^	1.29 × 10^−8^	NS	2.74 × 10^−11^
7	108,572	111,696 *	3125	116–119	NS	2.01 × 10^−11^	1.55 × 10^−11^	6.20 × 10^−5^	6.20 × 10^−5^	NS	NS
8	85,944 *	89,483	3540	091–094	NS	2.95 × 10^−12^	9.72 × 10^−11^	6.37 × 10^−9^	6.37 × 10^−9^	NS	NS
9	45,480	53,842	8363	050–060	NS	1.57 × 10^−8^	1.35 × 10^−5^	3.59 × 10^−9^	3.59 × 10^−9^	NS	6.29 × 10^−12^
10	102,225	103,207 *	983	110	NS	2.99 × 10^−4^	3.01 × 10^−5^	NS	NS	NS	2.96 × 10^−3^
11	149,094	151,035 *	1942	154–156	NS	3.17 × 10^−4^	1.88 × 10^−4^	NS	NS	NS	NS

* = The actual breakpoint position is undetermined (it was most likely overprinted by a subsequent recombination event).

**Table 3:** Proteins and amino acids of LSDV/Yasothon/2021 affected by the 11 genomic recombination events as predicted by RDP4.

Event	ORF	Protein	Amino Acid Differences from the LSDV Strain SIS-Lumpyvax Vaccine (Format: YST/Protein Position/SIS)
1	075	RNA polymerase-associated protein	V324A
	076	late transcription factor VLTF-4	V64A, D96DNDN, N101D, D102N, D151G
	079	mRNA capping enzyme, large subunit	I206T, D295N, T374P
	080	Virion protein	I26M, T93I
	081	Virion protein	H17N, S227N
	082	Uracil DNA glycosylase	R54Q
	083	NTPase; DNA replication	S3G, S49T, G106D, I135M, I253L, N663K, I708T
	084	Early transcription factor VETF transmembrane	L353V, D581N
	085	RNA polymerase subunit RPO18	M136T
	086a	*mut* T motif	E121D, L191F, 207 extension 3 amino acid residues due to frameshift mutation
	087a	*mut* T motif; gene expression regulator	D12G, V46I,196 extension 53 amino acid residues due to frameshift mutation, S199F, N200L
	088	NPH-I; transcription termination factor	V24I
	089	mRNA capping enzyme, small subunit; VITF	V171I
2	1	A52R-like family protein, SP	D129N, I144M
	3	ER-localised apoptosis regulator, SP, TM	S93T, A100S
	5	IL-10, SP, TM	I14IF, A24V, I25V
	6	IL-1 receptor, SP	F13L, S61L, AS94A, I111S, S216N
	7	IFN-g	Q58K, S200T, N295D
3	101	Virion core protein P4a	E223D
	102	TM	A61N, L115S, T162A
	103	Virion core protein	T50N, P72T, S89G
4	133	DNA ligase-like protein	V165I, D200E, S275N, L312S, I344T, D347N, S514A
5	146	Phospholipase D-like protein	T285S
	147	Ankyrin repeat protein	I487M
	148	Ankyrin repeat protein	G40S, G51D, I102L, N167D, E169D, V351M, K361Q, C397Y, K413E, A418T, N439S.
	149	Serpin-like protein CDS	K139R
6	027	EEV maturation protein	I440F, D328N, K197R, 153D, A61V
	028	Palmitylated EEV membrane lycoprotein	G263S, A135T
	030	Hypothetical protein	H34N
7	116	RNA polymerase subunit (RPO132)	N1154S
	118	Hypothetical protein, TM	I10V
	119	RNA polymerase subunit, RPO35	A291S
8	093	Hypothetical protein	D60N
	094	Virion core protein P4b, TM	D93N, F607L
9	050	Metalloprotease, virion morphogenesis	NT376T
	054	Hypothetical protein	S54G, M184I
	056	Hypothetical protein	K171R, N174D
	057	Virion core protein, TM	V372I
	059	Myristylated protein	Q125K
10	110	DNA helicase; transcriptional elongation factor	A64T, R100L, L344F
11	154	ER-localised apoptosis regulator	S93T, A100S
	156	A52R-like family protein, SP	D129N, I144M

The amino acid changes existing in the genome of China/GD01/2020 (MW355944) are underlined; the other 17 proteins involved in these major predicted recombination events without amino acid differences are not listed in this table. SP, N-terminal signal peptide; TM, transmembrane.

## Data Availability

The DNA sequences generated and used in the analysis for this study are available in GenBank under accession numbers MZ579540-MZ579542, MZ615173, OK323153, OM250058-OM250060, and OM033705.

## Supplementary Materials

The following supporting information can be downloaded at: https://www.mdpi.com/article/10.3390/vetsci9100542/s1, Figure S1: Some cattle showed conjunctivitis (A) and lameness due to severe skin lesions on phalanges (B), while some calves died from severe clinical signs (C). The lesions also included the nodular reproductive tract (D), inflammatory subscapular lymph nodes (E), and trachea ulcers (F); Figure S2: The histopathological lesions of LSDV infected skin; Figure S3: Immunohistochemistry of an LSDV infected skin tissue; Figure S4: Virus isolation from a skin nodule obtained from cattle with *p32* PCR positive; Figure S5: Phylogenetic tree based on complete *GPCR* gene (A) and *RPO30* gene (B) of Thai LSDV sequences compared with 35 reference strains of CaPV; Table S1: Sequences of oligonucleotide primers used for amplification and detection of LSDV genes; Table S2: LSDV and CaPV reference sequences used in the analysis; Table S3: The index cases of LSD outbreaks in each province from March 2021 to May 2022; Table S4: Numbers of provinces with LSDV positive in each region from March 2021 to June 2022; Table S5: GenBank Accession numbers of five Thai LSDV sequences; Video S1: Distribution of LSD outbreaks in Thailand from March to July 2021.
