# Supplementary material for: Rapid Spread and Genetic Characterisation of a Recently Emerged Recombinant Lumpy Skin Disease Virus in Thailand
Source: Vet Sci. 2022 Sep 30;9(10):542. doi: 10.3390/vetsci9100542 (PMC9609959; doi:10.3390/vetsci9100542)
Supplement: Supplementary file 1 [file vetsci-09-00542-s001.zip › vetsci-1839521-supplementary.pdf]

veterinary sciences

MDPI

Supplemental Materials

Table S1. Sequences of oligonucleotide primers used for amplification and detection of LSDV genes.

Target genes	Primer/probe names	Sequences from 5′ to 3′	References
p32	CaPV-074F1	AAA ACG GTA TAT GGA ATA GAG TTG GAA	[28]
	CaPV-074R1	AAA TGA AAC CAA TGG ATG GGA TA	
	CaPV-074P1	FAM-TGG CTC ATA GAT TTC CT-MGBNFQ	
RPO30	Cp*RPO30*-OL1F	CAG CTG TTT GTT TAC ATT TGA TTT TT	[33]
	Cp*RPO30*-OL1R	TCG TAT AGA AAC AAG CCT TTA ATA GA	
	Cp*RPO30*-OL2F	TTT GAA CAC ATT TTA TTC CAA AAA G	
	Cp*RPO30*-OL2R	AAC CTA CAT GCA TAA ACA GAA GC	
GPCR	Cp*GPCR*-OL1F	TGAAAAATTAATCCATTCTTCTAAACA	[33]
	Cp*GPCR*-OL1R	TCATGTATTTTATAACGATAATGCAAA	
	Cp*GPCR*-OL2F	TTAGCGGTATAATCATTCCAAATA	
	Cp*GPCR*-OL2R	GCGATGATTATGATGATTATGAAGTG	
	Cp*GPCR*-OL3F	CACAATTATATTTCCAAATAATCCAA	
	Cp*GPCR*-OL3R	TGTACATGTGTAATTTTAATGTTCGTA	

Table S2. LSDV and CaPV reference sequences used in the analysis.

No.	GenBank ID	Virus	Name	Identity to Thailand (OM033705)	Identity to vaccine (KX764643)	Identity to Wildtype (AF325528)
1*	AF325528	LSDV	Neethling-2490	99.24	98.79	
2*	KX683219	LSDV	KSGP0240	99.24	98.79	100
3*	MN072619	LSDV	Kenya	99.24	98.93	100
4*	AF409137	LSDV	Neethling Warmbaths	99.17	98.73	99.84
5	MW656253	LSDV	280-KZN-RSA	99.19	98.9	99.87
6	MN642592	LSDV	Kubash-KAZ	99.17	98.82	99.87
7	MN995838	LSDV	pendik	99.15	98.72	99.82
8	MT643825	LSDV	210LSD-249/BUL	99.17	98.77	99.86
9	MT130502	LSDV	Neethling-RIBSP	97.62	96.49	98.31
10*	MH893760	LSDV	Russia/Dagestan	99.18	98.76	99.87
11*	AF409138	LSDV	Neethling vaccine LW 1959	99.41	100	98.79
12*	KX764643	LSDV	Neethling-SIS-Lumpyvax	99.41		98.79
13*	MG972412	LSDV	Cro2016	99.41	100	98.79
14*	KX764644	LSDV	Neethling-Herbivac	99.41	100	98.79
15*	KX764645	LSDV	Neethling-OBP	99.42	99.97	98.8
16*	MW656252	LSDV	Haden/RSA	99.3	99.88	98.68
17	MN636843	LSDV	148-GP-RSA-1997	99.43	99.95	98.82
18	MW435866	LSDV	SA-Neethling	99.5	99.83	98.89
19*	MH646674	LSDV	Russia/Saratov/2017	99.4	99.48	99.11
20*	MW355944	LSDV	China/GD01/2020	99.99	99.41	99.23
21*	MZ577076	LSDV	20L81/Bang-Thanh/VNM-2020	100	99.41	99.24
22	OM033705	LSDV	Thailand/Yasothon/2021		99.41	99.24
23*	MW732649	LSDV	HongKong/2020	99.82	99.36	99.17
24*	MT134042	LSDV	Russia/Udmurtiya/2019	99.4	99.31	99.4

25	MN072621	Goatpox	Vietnam	90.44	90.6	90.66
26	MN072622	Goatpox	Turkey	97.95	97.88	98.09
27	MN072623	Goatpox	Oman	85.43	85.36	85.54
28	AY077834	Sheeppox	NISKHI	85.08	84.99	85.19
29	MN072630	Sheeppox	Saudi Arabia	85.08	84.99	85.19
30	MN072631	Sheeppox	Turkey	85.09	85	85.19
31	MG000156	Sheeppox	Jaipur	97.82	97.72	97.95
32	MN072629	Sheeppox	Pendik	85.08	84.99	85.2
33	MN072628	Sheeppox	Nigeria	85.09	84.99	85.2
34	MG000157	Sheeppox	Romanian Fenner	97.8	97.71	97.94
35	MN072626	Sheeppox	Abu Gharib	97.79	97.7	97.93
36	MN072627	Sheeppox	Saudi Arabia	97.8	97.71	97.94

* **Sequences aligned in phylogenetic trees of recombinant analysis.**

Table S3. The index cases of LSD outbreaks in each province from March 2021 to May 2022.

No.	Date of clinical sign reported (D/M/Y)	Regions	Provinces	Abbreviations
1	29/3/2021	Northeast	Roi Et	RET
2a	30/3/2021	Northeast	Ubon Ratchathani	UBN
2b	30/3/2021	Northeast	Surin	SRN
3a	31/3/2021	Northeast	Kalasin	KSN
3b	31/3/2021	Northeast	Nakhon Phanom	NPM
4	1/4/2021	Northeast	Buriram	BRM
5a	2/4/2021	Northeast	Maha Sarakham	MKM
5b	2/4/2021	Northeast	Aumnatcharoen	ACR
5c	2/4/2021	Northeast	Khon Kaen	KKN
6	5/4/2021	West	Ratchaburi	RBR
7	7/4/2021	Northeast	Sisaket	SSK
8	9/4/2021	Northeast	Yasothon	YST
9	14/4/2021	West	Phetchaburi	PBI
10	15/4/2021	West	Kanchanaburi	KRI
11	16/4/2021	Central	Nakhon Pathom	NPT
12	18/4/2021	Northeast	Mukdahan	MDH
13a	22/4/2021	West	Prachuap khiri Khan	PKN
13b	22/4/2021	West	Tak	TAK
14a	23/4/2021	Central	Suphan Buri	SPB
14b	23/4/2021	Northeast	Udon Thani	UDN
15	27/4/2021	Central	Sukhothai	STI
16	29/4/2021	Central	Lop Buri	LRI
17a	30/4/2021	Northeast	Nong Khai	NKI
17b	30/4/2021	South	Chumphon	CPN
18a	1/5/2021	North	Chiang Rai	CRI
18b	1/5/2021	Central	Pathum Thani	PTE
18c	1/5/2021	Northeast	Sakon Nakhon	SNK
18d	1/5/2021	Central	Angthong	ATG
19	4/5/2021	North	Lampang	LPG
20	5/5/2021	Northeast	Chaiyaphum	CPM
21	9/5/2021	Central	Phichit	PCT
22a	10/5/2021	Northeast	Nongbua Lumphoo	NBP
22b	10/5/2021	Northeast	Beung Kan	BKN
23	12/5/2021	Central	Phitsanulok	PLK
24a	13/5/2021	Central	Phra Nakhon Si Ayuttaya	AYA
24b	13/5/2021	Northeast	Sa Kaew	SKW
24c	13/5/2021	Northeast	Loei	LEI

25a	17/5/2021	Central	Phetchabun	PNB
25b	17/5/2021	North	Nan	NAN
26a	18/5/2021	Northeast	Nakhon Ratchasima	NMA
26b	18/5/2021	Central	Saraburi	SRI
27a	19/5/2021	North	Phayao	PYO
27b	19/5/2021	Central	Uthai Thani	UTI
28	20/5/2021	North	Phrae	PRE
29	21/5/2021	East	Cha Choeng Sao	CCO
30	22/5/2021	East	Chon Buri	CBI
31a	23/5/2021	Central	Samut Prakan	SPK
31b	23/5/2021	East	Prachin Buri	PRI
32	25/5/2021	Central	Sing Buri	SBR
33	27/5/2021	Central	Bangkok	BKK
34	28/5/2021	Central	Chai Nat	CNT
35	5/6/2021	North	Lumphun	LPN
36	9/6/2021	South	Yala	YLA
37a	16/6/2021	North	Chiang Mai	CMI
37b	16/6/2021	East	Chanthaburi	CTI
38	20/6/2021	Central	Nakhon Sawan	NSN
39	21/6/2021	North	Uttaradit	UTT
40	24/6/2021	Central	Samut Sakhon	SKN
41	26/6/2021	South	Song Khla	SKA
42	29/6/2021	Central	Samut Songkhram	SKM
43	2/7/2021	Central	Kamphaeng Phet	KPT
44	5/7/2021	South	Krabi	KBI
45	17/7/2021	South	Phatthalung	PLG
46	29/7/2021	Central	Nakhon Nayok	NYK
47	17/8/2021	Central	Nonthaburi	NBR
48	26/8/2021	South	Surat Thani	STN
49	7/9/2021	East	Trat	TRT
50	19/10/2021	South	Nakhon Si Thammarat	NST
51	11/2/2022	South	Trang	TRG
52	30/5/2022	South	Phuket	PHK

Table S4. Numbers of provinces with LSDV positive in each region from March 2021 to June 2022.

Years	Months	Regions						Total Provinces
		North	Northeast	Central	East	West	South	
**2021**	**March**	0	5	0	0	0	0	5
2021	April	0	9	4	0	5	1	19
2021	May	5	6	12	4	0	0	27
2021	June	4	0	2	1	0	2	9
2021	July	0	0	2	0	0	2	4
2021	August	0	0	1	0	0	1	2
2021	September	0	0	0	1	0	0	1
2021	October	0	0	0	0	0	1	1
2021	November	0	0	0	0	0	0	0
2021	December	0	0	0	0	0	0	0
2022	January	0	0	0	0	0	0	0
2022	February	0	0	0	0	0	1	0
2022	March	0	0	0	0	0	0	0
2022	April	0	0	0	0	0	0	0
2022	May	0	0	0	0	0	1	1
2022	June	0	0	0	0	0	0	0

Total affected provinces	9	20	21	6	5	9	70
No. provinces per region	9	20	22	7	5	14	77

Table S5. GenBank Accession numbers of five Thai LSDV sequences.

No.	Isolates	Accession Numbers		
		RPO30	*GPCR*	Whole genome
1	LSDV/Thailand/Roi Et/2021	MZ579540	OM250058	-
2	LSDV/Thailand/Sisaket/2021	MZ579541	OM250059	-
3	LSDV/Thailand/Lopburi/2021	MZ579542	OM250060	-
4	LSDV/Thailand/Kanchanaburi/2021	MZ615173	OK323153	-
5	LSDV/Thailand/Yasothon/2021	-	-	OM033705

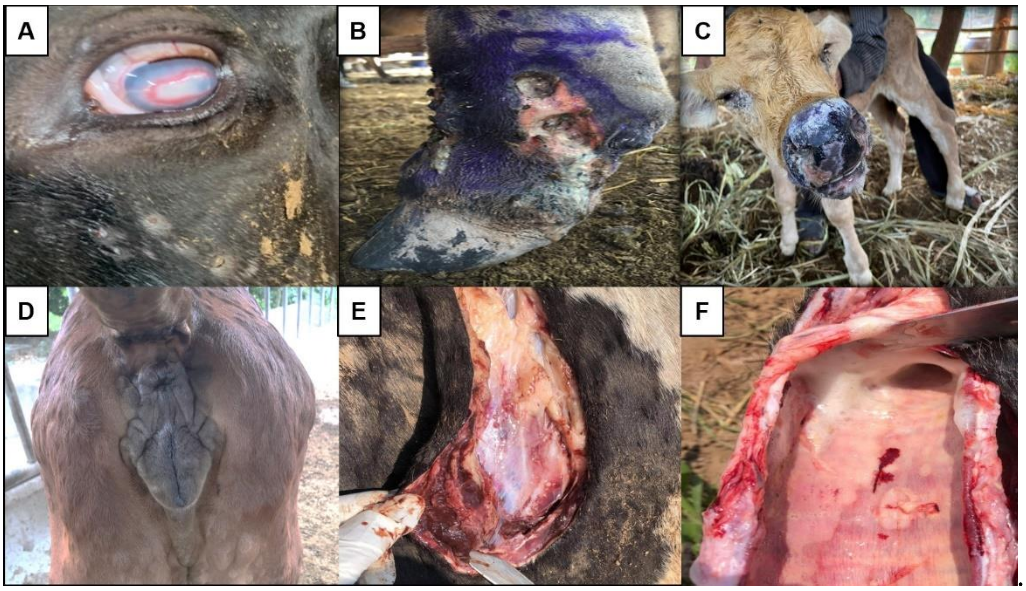

Figure S1. Some cattle showed conjunctivitis (A) and lameness due to severe skin lesions on phalanges (B), while some calves died from severe clinical signs (C). The lesions also included the nodular reproductive tract (D), inflammatory subscapular lymph nodes (E), and trachea ulcers (F).

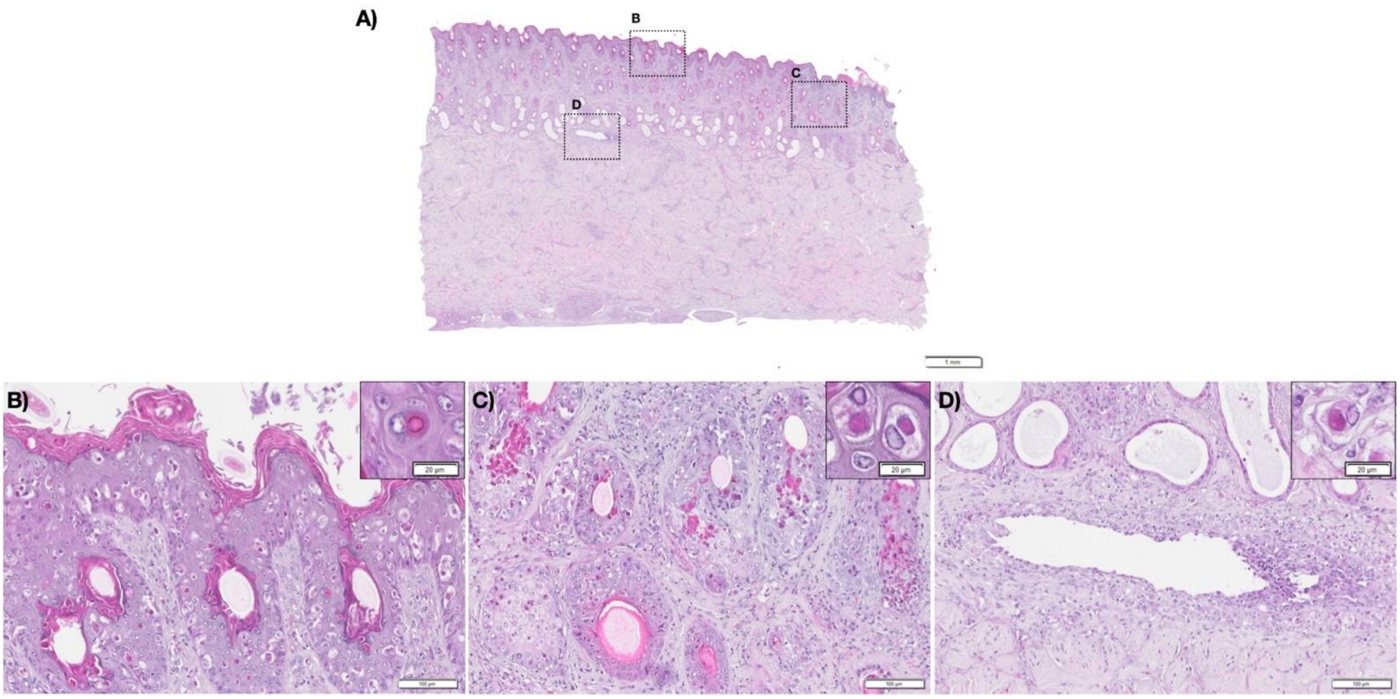

Figure S2. The histopathological lesions of LSDV infected skin. At low magnification, the LSD lesions diffusely distributed in the whole top layer of the tissue section comprising epidermis, and deeply into deep dermis and muscle tissue (A). The high magnification views (dashed boxes) depict epidermis (B), hair follicle and sebaceous glands (C), and blood vessels (D). The intracytoplasmic inclusion bodies were a brighten-pink, round to oval shape in cytoplasm of epidermal cells including infiltrating macrophages as shown in the insets.

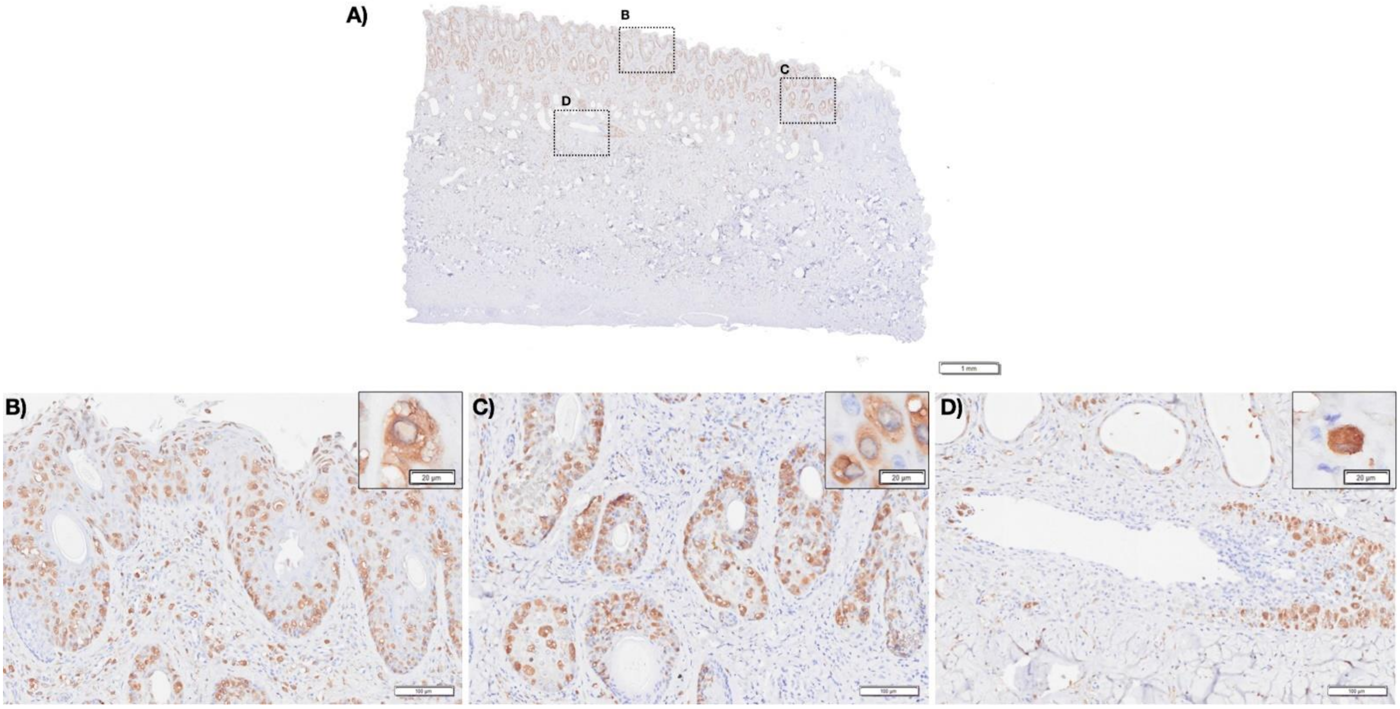

Figure S3. Immunohistochemistry of an LSDV infected skin tissue. The brown staining LSDV positive cells abundantly diffused throughout the epidermis and dermis layers of the skin (A) consistent with the histopathological findings in Figure S2. The high magnification dashed boxes (B–D) are

corresponding areas in the consecutive tissue section shown in the Figure S2. The LSDV antigens accumulated in cytoplasm of the infected cells as shown in the insets.

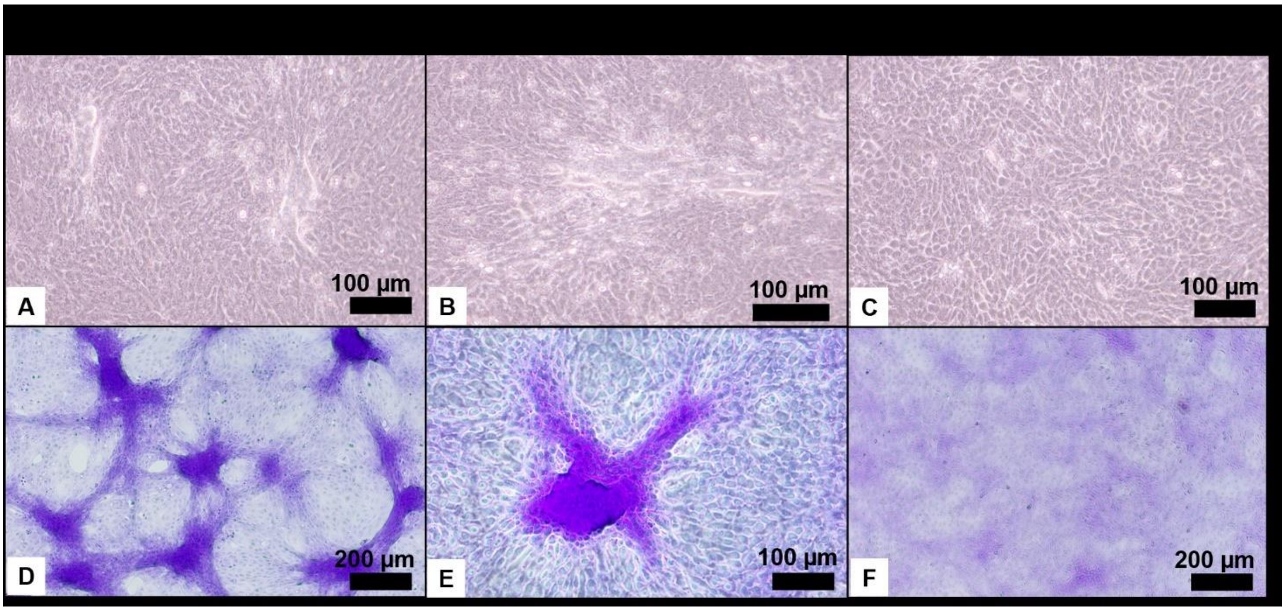

Figure S4. Virus isolation from a skin nodule obtained from cattle with *p32* PCR positive. (A-B) The CPE was developed at 3 dpi in MDBK cells of the third passage. (C) MBDK cell culture control without CPE. (D-E) The CPE was clearly demonstrated by crystal violet staining. (F) No CPE was observed in MBDK cell control.

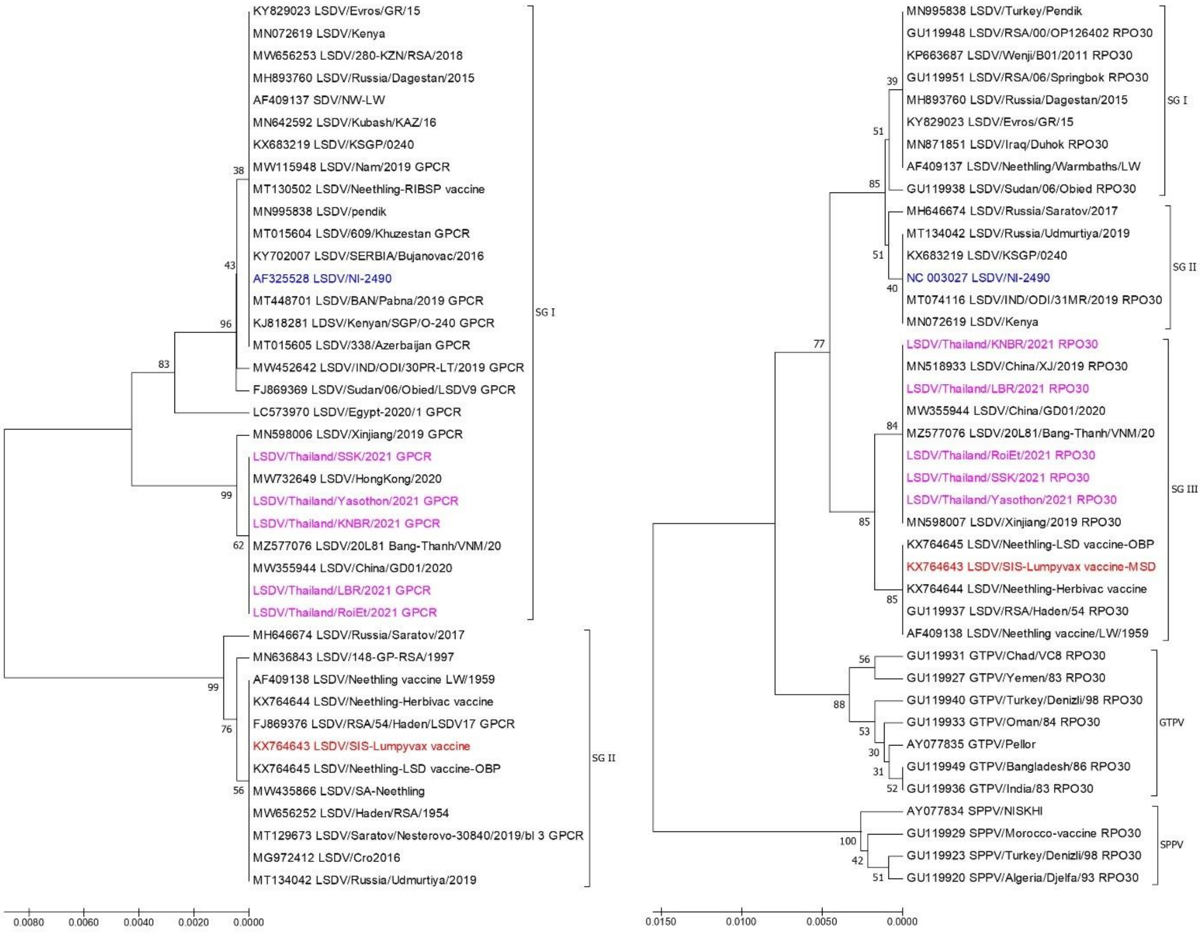

Figure S5. Phylogenetic tree based on complete *GPCR* gene (A) and *RPO30* gene (B) of Thai LSDV sequences compared with 35 reference strains of CaPV. The trees were analyzed by UPGMA method with 1,000 bootstraps. The evolutionary distances were computed using the Tamura 3-parameter model. Evolutionary analyses were conducted in MEGA 7. *GPCR* sequences of Thai LSDV isolates (pink) were in subgroup I with LSDV/NI-2490 (blue), while SIS-Lumpyvax vaccine was in subgroup II (red). RPO30 sequences of Thai LSDV isolates were in subgroup III with SIS-Lumpyvax vaccine, while LSDV/NI-2490 strain was in subgroup II.

Video S1. Distribution of LSD outbreaks in Thailand from March to July 2021.
